# Effects of High-Intensity Interval Training in Older Adults: A Systematic Review of Randomized and Non-Randomized Intervention Studies

**DOI:** 10.3390/jcm15176701

**Published:** 2026-08-29

**Authors:** Paula Moreira, Sérgio Ferreira, Nuno Domingos Garrido, António M. Monteiro, Pedro Afonso, Alexandra Malheiro, Joana Ribeiro, Pedro Forte

**Affiliations:** 1 Department of Sport, Exercise and Health Sciences, University of Trás-os-Montes e Alto Douro, 5000-801 Vila Real, Portugal; srafael.ferreira456@gmail.com (S.F.); ndgarrido@gmail.com (N.D.G.); 2 Research Center in Sports Sciences, Health Sciences and Human Development (CIDESD), 5000-801 Vila Real, Portugal; 3 Research Centre for Active Living and Wellbeing (Livewell), Instituto Politécnico de Bragança, 5300-253 Bragança, Portugalpmvafonso@gmail.com (P.A.); alexandra.malheiro@iscedouro.pt (A.M.); pedromiguel.forte@iscedouro.pt (P.F.); 4 Department of Sports Sciences, Instituto Politécnico de Bragança, 5300-252 Bragança, Portugal; 5 Department of Sports, Higher Institute of Educational Sciences of the Douro, 4560-708 Penafiel, Portugal; joana.ribeiro@iscedouro.pt; 6 Biosciences Higher School of Elvas, Polytechnic Institute of Portalegre, 7300-110 Portalegre, Portugal; 7 Life Quality Research Center (LQRC-CIEQV), 2001-964 Santarém, Portugal

**Keywords:** exercise prescription, aerobic interval training, ageing, cardiorespiratory fitness, functional capacity, quality of life

## Abstract

**Background:** High-intensity interval training (HIIT) is a time-efficient exercise strategy, but its effects in older adults remain incompletely established. **Objective:** Our objective is to synthesize evidence on the effects of HIIT and aerobic interval training (AIT) on cardiorespiratory, metabolic, functional, cognitive, psychological, and quality-of-life outcomes in older adults. **Methods:** This systematic review was reported in accordance with PRISMA 2020. PubMed, Scopus, and Web of Science were searched through 13 May 2026 for randomized and non-randomized longitudinal intervention studies involving participants with a mean or median age ≥ 60 years. Titles/abstracts and full texts were screened by one reviewer, with uncertain eligibility resolved through discussion with the supervisory team. Data were extracted by one reviewer and independently verified by a second reviewer. The review was retrospectively registered in INPLASY (INPLASY202660027). **Results:** Twenty-five eligible reports were included, representing approximately 2818 unique participants. The most consistent findings concerned cardiorespiratory fitness, with improvements in VO_2_peak and exercise tolerance reported across several populations. Selected functional and metabolic benefits were also observed, whereas cognitive, psychological, and quality-of-life findings were more limited and heterogeneous. HIIT produced comparable or greater improvements than moderate-intensity continuous training in some studies, but overall superiority was not established. Feasibility and adherence were generally favourable, although adverse-event reporting was inconsistent. **Conclusions:** HIIT/AIT may improve cardiorespiratory fitness and selected functional outcomes in older adults. However, confidence in these findings is limited by heterogeneity in populations, protocols, and outcomes, together with the predominance of risk-of-bias concerns across the included evidence. The available findings do not establish overall superiority, comparative safety, or an optimal HIIT/AIT prescription.

## 1. Introduction

Population aging represents one of the major public health challenges of the twenty-first century and is accompanied by a progressive decline in cardiovascular, neuromuscular, metabolic, and cognitive function [1]. These physiological changes contribute to frailty, loss of functional independence, and increased morbidity and mortality [2]. Cardiorespiratory fitness (CRF), commonly assessed through maximal or peak oxygen uptake (VO_2_peak), is recognized as one of the strongest predictors of healthy aging, functional capacity, and longevity in older adults [3]. Aging is also associated with sarcopenia, reduced muscular power, impaired balance, and mobility limitations, factors strongly linked to falls, disability, and institutionalization [4]. Consequently, preserving physical function and physiological reserve has become a major focus of geriatric medicine and exercise science [5].

Regular physical exercise is widely recognized as one of the most effective non-pharmacological strategies to attenuate age-related decline and improve health outcomes in older adults. International guidelines recommend that older individuals engage in regular aerobic and muscle-strengthening activities to maintain physical and cognitive function and reduce chronic disease risk [6]. Nevertheless, physical inactivity remains highly prevalent in this population, particularly among individuals with chronic diseases or functional limitations. Common barriers to exercise participation include lack of time, reduced motivation, fear of injury, advanced age, and poor health status [7]. Historically, conditions such as heart failure were considered contraindications to exercise, but this perspective has changed substantially, with exercise-based rehabilitation now strongly recommended across several cardiometabolic and neurological conditions [8].

In this context, high-intensity interval training (HIIT) has emerged as a time-efficient and effective exercise strategy for older populations [9,10,11]. HIIT is characterized by repeated bouts of vigorous-to-near-maximal exercise interspersed with periods of recovery [12]. Compared with moderate-intensity continuous training (MICT), HIIT has demonstrated comparable or superior improvements in VO_2_peak, endothelial function, insulin sensitivity, vascular health, and muscular performance across different populations and clinical settings [13]. Additionally, HIIT may be perceived as more enjoyable and less monotonous than continuous exercise, potentially improving long-term adherence [14]. Importantly, HIIT protocols can be adapted to different functional capacities and clinical contexts, ranging from supramaximal cycle ergometer protocols to low-volume and home-based interventions [7,15].

The application of HIIT has progressively expanded to clinically complex older populations characterized by multimorbidity [16,17]. Conditions such as coronary artery disease (CAD), heart failure (HF), chronic obstructive pulmonary disease (COPD), type 2 diabetes mellitus (T2DM), stroke, and HIV-associated accelerated aging are frequently associated with exercise intolerance, autonomic dysfunction, chronic inflammation, skeletal muscle impairment, and reduced quality of life [18,19,20,21,22]. Emerging evidence suggests that HIIT may improve cardiorespiratory fitness, metabolic regulation, vascular function, mobility, and psychological well-being [23,24,25] across different chronic-disease and rehabilitation settings [26].

Despite the growing interest in HIIT, important limitations remain in the literature. Many studies have focused on younger populations, acute responses, or highly controlled settings, limiting the applicability of findings to broader aging populations [9,10,11]. Furthermore, HIIT protocols differ substantially regarding interval duration, intensity prescription, exercise modality, supervision, and training volume, making comparisons across studies difficult [16]. Safety and tolerability also remain important considerations, particularly in frail or multimorbid older adults. However, recent evidence supports the feasibility and safety of appropriately supervised HIIT even in sedentary older adults and patients with cardiovascular or metabolic disease [15,27].

Recent systematic reviews and meta-analyses have strengthened the evidence supporting HIIT in older adults. Men et al. [28] quantitatively examined the effects of HIIT on body composition, cardiopulmonary function, and metabolic indicators, whereas Sert et al. [29] focused primarily on cardiometabolic health, functional capacity, and quality of life. Although these reviews provide important quantitative evidence, their scope was concentrated predominantly on cardiopulmonary, metabolic, anthropometric, and selected functional or quality-of-life outcomes.

The present review was designed to provide a complementary and updated synthesis of the evidence by integrating a broader range of outcomes, including cardiorespiratory and vascular function, metabolic health, muscular and functional performance, cognitive and psychological outcomes, health-related quality of life, and intervention feasibility, adherence, and safety. It additionally considers the substantial diversity of HIIT/AIT modalities and prescription characteristics across apparently healthy older adults and clinically complex aging populations, while incorporating evidence identified through May 2026. Accordingly, this systematic review aimed to determine the effects of HIIT and AIT, compared with MICT, usual care, non-exercise control, or alternative exercise interventions, across these health and functional domains. For the purposes of the present review, older adults were operationally defined as populations with a mean or median age of at least 60 years, or studies reporting separately extractable data for participants aged ≥60 years. This operational threshold was selected in accordance with the United Nations standard adopted in the World Health Organization Active Ageing framework, which uses 60 years to describe older people [30].

## 2. Methods

### 2.1. Protocol and Registration

This systematic review was reported in accordance with the Preferred Reporting Items for Systematic Reviews and Meta-Analyses (PRISMA 2020) statement, which guided all stages of the review process, including database searching (Table S1), study selection, data extraction, and evidence synthesis (Table S2) [31]. The review was designed to comprehensively examine and synthesize the available evidence on the effects of high-intensity interval training (HIIT) on physiological, cardiorespiratory, metabolic, functional, cognitive, and health-related quality-of-life outcomes in healthy and clinically complex older adults.

Only randomized controlled trials (RCTs) and non-randomized longitudinal clinical intervention studies investigating chronic adaptations to HIIT were considered eligible for inclusion. Given the substantial methodological and clinical heterogeneity observed across eligible studies, particularly regarding participant characteristics, clinical conditions, HIIT protocol design, intervention duration, and outcome measures, a narrative synthesis approach was adopted in lieu of a quantitative meta-analysis. The review was retrospectively registered with the International Platform of Registered Systematic Review and Meta-analysis Protocols (INPLASY; INPLASY202660027; permanent DOI: 10.37766/inplasy2026.6.0027) on 5 June 2026. Registration occurred after commencement of the review process and should therefore not be interpreted as prospective registration. The INPLASY record was updated on 31 July 2026 to remove unrelated CrossFit-related text that had been inadvertently retained in the original PICO and subgroup-analysis fields and to restate these elements in accordance with the HIIT/AIT review question.

Additional methodological changes were introduced during peer review and are reported here as deviations from the registered record. First, the previous condition-specific lower age threshold of ≥50 years was removed and replaced by a uniform criterion requiring a reported mean or median age ≥ 60 years or separately extractable data for participants aged ≥60 years. Reapplication of this criterion reduced the eligible evidence base from 34 to 25 reports. Second, the PEDro-based methodological quality assessment specified in the registration was replaced by design-specific risk-of-bias assessment using RoB 2 for randomized studies and ROBINS-I for non-randomized intervention studies. Third, the narrative synthesis was refined to distinguish within-group changes from between-group effects and from evidence of superiority over active comparators. These changes were made, respectively, to improve consistency of population eligibility, apply design-appropriate risk-of-bias methods, and reduce overinterpretation of favorable within-group findings.

### 2.2. Eligibility Criteria

Studies involving healthy older adults and clinically complex populations presenting with age-related chronic conditions were considered eligible. Clinical populations included individuals diagnosed with coronary artery disease, heart failure, ischaemic stroke, chronic obstructive pulmonary disease, type 2 diabetes mellitus, osteoarthritis, pulmonary embolism, and HIV-associated accelerated aging. Eligible studies were required to report a mean or median participant age of ≥60 years or to provide separately extractable data for participants aged ≥60 years. For mixed-age samples, eligibility was determined using the reported study-level mean or median age when age-stratified data were not available. No minimum proportion of participants aged ≥60 years was required because individual-level age distributions or the proportion of participants above specific age thresholds were not consistently reported across the eligible studies. Studies with a reported mean or median age < 60 years were eligible only when data for participants aged ≥60 years could be separately extracted. No lower age threshold was permitted on the basis of clinical condition or presumed accelerated aging.

Eligible interventions comprised structured high-intensity interval training (HIIT) or aerobic interval training (AIT) protocols, defined by repeated bouts of vigorous-to-near-maximal effort interspersed with periods of active or passive recovery. Home-based, aquatic, resistance-based, and combined HIIT modalities were considered eligible provided the intervention design clearly adhered to the conceptual and operational characteristics of HIIT.

HIIT/AIT interventions were required to include repeated high-intensity exercise bouts prescribed or monitored using objective or subjective intensity markers, including percentage of peak oxygen uptake, maximal heart rate, heart-rate reserve, peak power output, maximal aerobic speed, workload, or rating of perceived exertion.

Eligible comparator conditions included moderate-intensity continuous training (MICT), standard care, non-exercise control groups, and alternative structured exercise interventions. Supervised or unsupervised rehabilitation-based HIIT/AIT programs were eligible when the intervention involved a structured repeated-session training program and met the operational definition of HIIT/AIT.

Studies reporting at least one physiological, cardiorespiratory, metabolic, functional, cognitive, psychological, or health-related quality-of-life outcome were eligible. Outcomes of interest included, but were not limited to, peak oxygen uptake (VO_2_peak), resting and exercise blood pressure, endothelial function, insulin sensitivity, lipid profile, body composition, muscular strength, gait performance, postural balance, cognitive function, depressive symptomatology, health-related quality of life, and exercise adherence.

Only randomized controlled trials and longitudinal clinical intervention studies published in peer-reviewed journals were included. Studies investigating exclusively acute or within-session physiological responses to exercise were excluded, as the present review focused specifically on chronic training-induced adaptations. The following study types were also excluded: systematic reviews, meta-analyses, narrative reviews, study protocols, observational studies, methodological investigations, case reports, conference abstracts, animal studies, and in vitro investigations. Additionally, studies exclusively involving children, adolescents, young adults, or competitive athletes were excluded. Studies lacking a clearly defined HIIT or AIT intervention were also excluded from the review.

An additional sensitivity search was conducted without study-design filters to ensure that eligible non-randomized longitudinal intervention studies had not been missed. This search identified no additional eligible reports. Complete database-specific search strategies are provided in Supplementary Table S1.

### 2.3. Search Strategy

The search strategy was developed to maximize both sensitivity and specificity, combining controlled vocabulary terms and free-text keywords related to three conceptual domains: high-intensity interval training, older adult populations, and randomized or longitudinal clinical study designs. Boolean operators (“AND” and “OR”) were employed to combine search terms within and across domains, and the strategy was independently adapted to the indexing conventions and field-specific syntax of each database.

The PubMed search strategy was structured as follows:

(“High-Intensity Interval Training”[MeSH] OR “high-intensity interval training”[tiab] OR HIIT[tiab] OR “high intensity interval training”[tiab] OR “high intensity interval exercise”[tiab] OR “aerobic interval training”[tiab]) AND (“Aged”[MeSH] OR “older adults”[tiab] OR elderly[tiab] OR aged[tiab] OR seniors[tiab] OR aging[tiab] OR “older people”[tiab]) AND (“Randomized Controlled Trial”[Publication Type] OR “Clinical Trial”[Publication Type] OR “randomized controlled trial”[tiab] OR “randomised controlled trial”[tiab] OR RCT[tiab] OR “clinical trial”[tiab]).

Equivalent search strings were constructed for Scopus using TITLE-ABS-KEY field tags and for the Web of Science Core Collection using TS (Topic) field tags, with appropriate syntactic adaptations. Database-specific filters were applied where available, including restrictions to human studies, clinical and randomized controlled trial publication types, and English-language publications.

### 2.4. Study Selection

A comprehensive and systematic literature search was conducted across three major electronic bibliographic databases: PubMed, Scopus, and the Web of Science Core Collection. These databases were selected for their extensive and complementary coverage of the biomedical, clinical, sports science, and exercise physiology literature, thereby maximizing the likelihood of identifying all relevant published evidence. The search was executed on 13 May 2026.

The initial search yielded 614 records in PubMed, 1382 records in Scopus, and 374 records in the Web of Science Core Collection, totalling 2370 records prior to deduplication. Following the application of database-specific filters and systematic removal of duplicate records, 1326 unique records were retained for subsequent title and abstract screening.

All retrieved references were imported into Zotero (version 7.0) for systematic organization and duplicate management. To minimize the risk of relevant studies being omitted, the reference lists of all included studies and pertinent review articles were manually screened for potentially eligible investigations not captured by the electronic search strategy.

Following retrieval from all databases, records were exported and consolidated in Zotero, where duplicates were removed before screening. The selection process was conducted in two sequential phases in accordance with PRISMA 2020 recommendations. Studies were excluded at full-text review if they did not involve older adults or clinically relevant aging populations, lacked a clearly defined HIIT or AIT intervention, investigated exclusively acute or within-session physiological responses, or did not employ a longitudinal intervention design.

**Figure 1:**
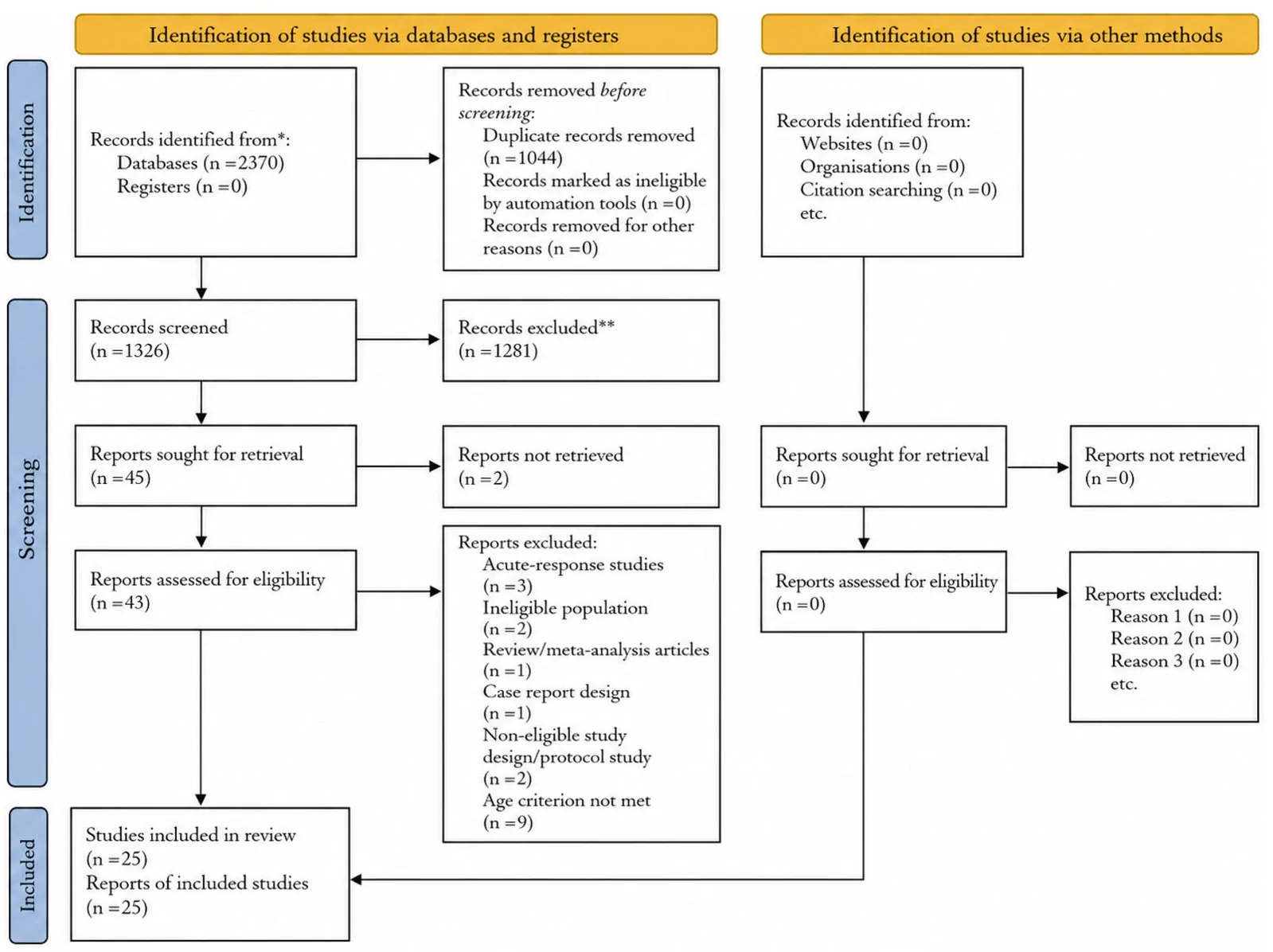
PRISMA 2020 flow diagram of the study selection process. * Web of Science, PubMed and Scopus. ** excluded by the authors revision.

The screening and full-text eligibility assessment were conducted by the primary reviewer, with ambiguous cases resolved through discussion with the supervisory team. The complete study selection process, including the number of records identified, screened, excluded, and included at each stage, is summarized in the PRISMA 2020 flow diagram presented in Section 3 (Figure 1).

### 2.5. Data Extraction

Data extraction was performed systematically using a standardized extraction framework developed a priori for the purposes of this review. Information extracted from each included study comprised authorship, year of publication, country of origin, study design, sample size, participant characteristics, clinical condition, age, sex distribution, intervention characteristics, comparator conditions, intervention duration, training frequency, exercise intensity prescription method, exercise modality, and primary outcome measures.

Additional data pertaining to physiological, cardiorespiratory, metabolic, functional, cognitive, psychological, and health-related quality-of-life outcomes were extracted where reported. Information concerning exercise adherence, feasibility, tolerability, and adverse events was systematically recorded to support the clinical interpretation and practical applicability of HIIT interventions across healthy and clinically complex older populations.

The standardized extraction form was pilot-tested using 25 eligible reports and refined before full data extraction. Data extraction was performed by one reviewer and independently verified for accuracy and completeness by a second reviewer. Discrepancies were resolved through discussion and consultation with a third member of the review team when necessary.

Outcomes explicitly designated by the study authors as primary were recorded as primary outcomes. When no primary outcome was identified, the principal outcome corresponding to the objective of the individual report was selected. Secondary outcomes were extracted when they contributed to the prespecified domains of the review.

The primary post-intervention time point was used for the main synthesis. Follow-up assessments were reported separately and were not combined with immediate post-intervention results. When several instruments assessed the same outcome domain, each instrument was extracted separately, while priority in the narrative synthesis was given to the instrument identified as primary by the study authors.

Missing or incompletely reported data were recorded as not reported and were not imputed by the review authors. Where numerical results were unavailable, findings were described using the information reported by the study authors. Multiple publications arising from the same parent trial were linked to prevent double counting of participants.

### 2.6. Risk of Bias Assessment

Risk of bias was assessed at the result level using design-specific tools. Randomized controlled trials were evaluated using the Cochrane Risk of Bias 2 tool (RoB 2), whereas non-randomized intervention studies were evaluated using the Risk Of Bias In Non-randomized Studies of Interventions tool (ROBINS-I). The crossover-specific version of RoB 2 was applied where appropriate [32,33].

For each report, the primary result specified by the study authors was selected for assessment. When no primary outcome was clearly identified, the principal result contributing to the present review at the end-of-intervention time point was assessed. Additional result-specific assessments were undertaken when distinct outcomes from the same report contributed to substantively different conclusions.

RoB 2 evaluates bias arising from the randomization process, deviations from the intended interventions, missing outcome data, measurement of the outcome, and selection of the reported result. Domain-level and overall judgments were categorized as low risk of bias, some concerns, or high risk of bias [33].

ROBINS-I evaluates bias due to confounding, selection of participants into the study, classification of interventions, deviations from intended interventions, missing data, measurement of outcomes, and selection of the reported result. Domain-level and overall judgments were categorized as low, moderate, serious, or critical risk of bias, or no information [32].

Risk-of-bias assessments were conducted independently by two reviewers. Disagreements were resolved through discussion and consensus, with consultation of a third reviewer when necessary.

### 2.7. Data Synthesis

Given the substantial clinical and methodological heterogeneity across the included studies, particularly in participant characteristics, clinical conditions, intervention modalities, HIIT/AIT protocols, comparator conditions, and outcome measures, quantitative pooling was considered inappropriate. Accordingly, a structured synthesis without meta-analysis was conducted.

Studies were grouped according to prespecified outcome domains: cardiorespiratory and physiological outcomes, metabolic outcomes, functional performance, cognitive and psychological outcomes, health-related quality of life, and feasibility and safety. Within each domain, findings were further considered according to population characteristics, intervention modality, comparator condition, and direction of effect. Where sufficient quantitative information was available, absolute or relative changes and between-group differences were considered alongside statistical significance and clinical relevance. Within-group improvements were distinguished from between-group effects and from evidence of superiority over active comparators.

To enhance transparency and facilitate interpretation across the heterogeneous evidence base, the findings were additionally summarized using tabular and graphical evidence-mapping approaches rather than pooled effect estimates.

Risk-of-bias judgments were incorporated into the interpretation of the evidence. Greater interpretative weight was given to randomized results judged at low risk of bias. Results judged at high risk in RoB 2 or at serious or critical risk in ROBINS-I were retained in the descriptive synthesis but were not considered sufficient, in isolation, to support firm conclusions regarding efficacy, superiority, safety, or clinical effectiveness.

The direction of findings was therefore interpreted alongside study design, comparator condition, sample size, distinction between within-group and between-group effects, and risk-of-bias judgment.

## 3. Results

### 3.1. Study Selection

The database search identified 2370 records across PubMed (*n* = 614), Scopus (*n* = 1382), and Web of Science (*n* = 374). After duplicate removal, 1326 records remained for title and abstract screening. Forty-three reports underwent full-text assessment, of which 18 were excluded because of acute-response study design (*n* = 3), ineligible population (*n* = 2), review or meta-analysis design (*n* = 1), case report design (*n* = 1), protocol or other non-eligible study design (*n* = 2), or failure to meet the predefined age criterion (*n* = 9). Twenty-five eligible reports were included in the qualitative synthesis. Publications arising from the same parent trial were identified and treated as related reports rather than as independent participant samples. The complete study-selection process is presented in Figure 1.

### 3.2. Characteristics of the Included Studies

A total of 25 studies were included in the final qualitative synthesis, comprising predominantly randomized controlled trials (RCTs) conducted across apparently healthy older adults and clinically complex aging populations. Sample sizes ranged from fewer than 20 participants in pilot studies to 1567 community-dwelling older adults.

Populations included apparently healthy or sedentary older adults and individuals with cardiovascular disease, stroke, COPD, type 2 diabetes, osteoarthritis, pulmonary embolism, intellectual disability, and HIV.

Considerable heterogeneity was observed across HIIT and AIT protocol design, exercise modality, intervention duration, and intensity prescription method. Intervention duration ranged from 4 weeks to 5 years.

**Figure 2:**
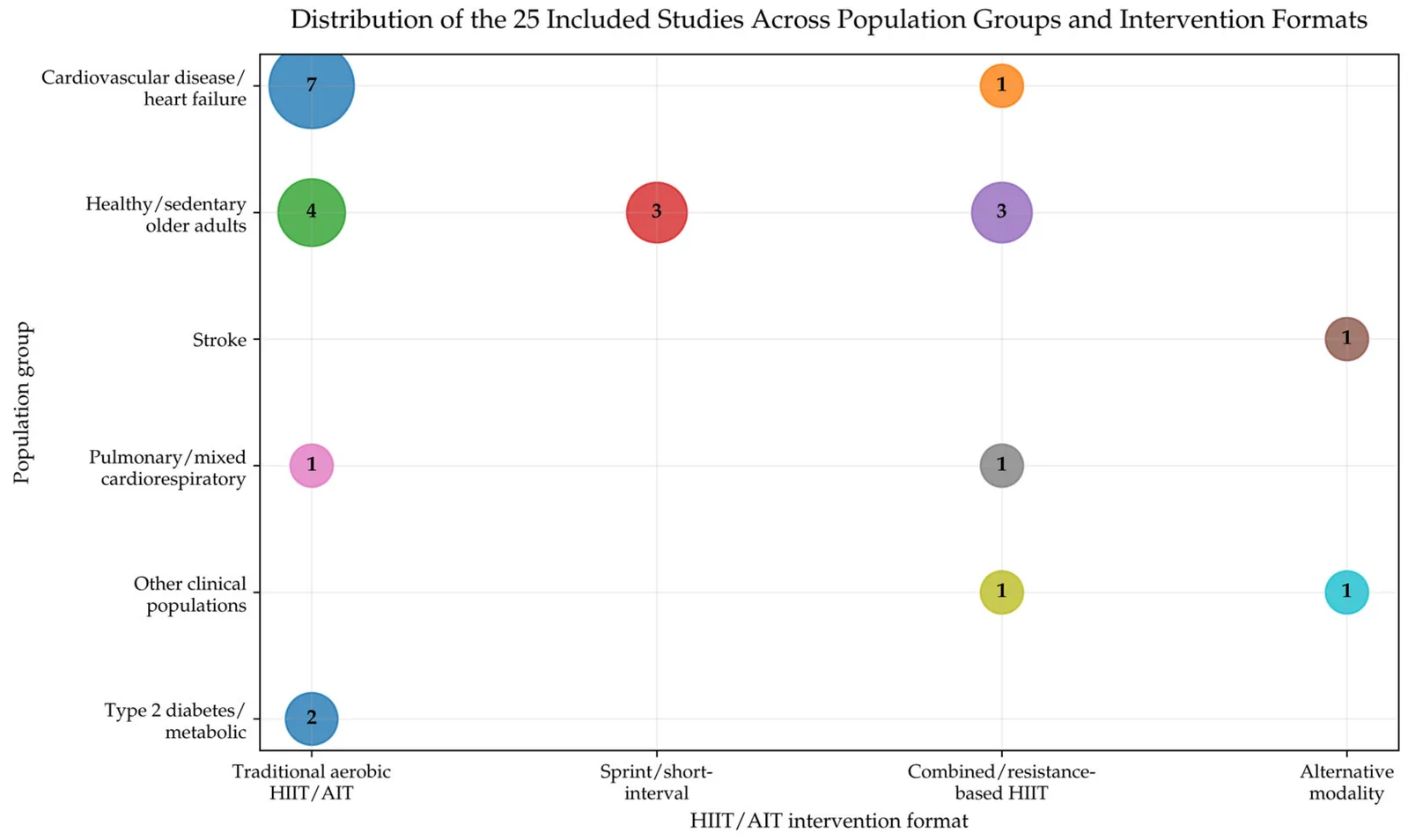
Distribution of the 25 included studies across population groups and HIIT/AIT intervention formats. Bubble size and labels indicate the number of studies in each population-intervention category. Categories represent descriptive groupings used to visualize the heterogeneity of the evidence base and do not imply statistical pooling.

Comparator conditions included moderate-intensity continuous training (MICT), standard medical care, non-exercise control groups, residential rehabilitation programmes, and alternative structured exercise modalities. Outcomes spanned cardiorespiratory, metabolic, functional, cognitive, psychological, and quality-of-life domains. Detailed characteristics of all included studies are presented in Table 1, and the distribution of studies across population groups and intervention formats is illustrated in Figure 2.

### 3.3. Participant Characteristics

The included studies involved heterogeneous samples of apparently healthy older adults and individuals with cardiovascular, metabolic, neurological, pulmonary, and musculoskeletal conditions. Participant age varied across studies. All retained reports met the revised operational age criterion on the basis of a reported mean or median age ≥ 60 years or separately extractable data for participants aged ≥60 years. Some eligible studies included mixed-age samples containing participants younger than 60 years; in these cases, eligibility was based on the reported study-level mean or median age because age-stratified participant data were not consistently available. Most studies included both sexes, although some cardiac rehabilitation cohorts were predominantly male and one metabolic study included women only.

The evidence base was concentrated primarily in apparently healthy or sedentary older adults and cardiovascular populations, which accounted for the majority of included studies. Considerably fewer studies investigated type 2 diabetes mellitus, pulmonary conditions, stroke, and other clinical populations. Clinical cohorts generally presented with reduced baseline cardiorespiratory fitness, exercise tolerance, muscular function, mobility, or balance. Detailed participant characteristics are presented in Table 2.

### 3.4. Characteristics of HIIT Protocols

Substantial heterogeneity was observed across the HIIT/AIT interventions in terms of exercise modality, interval structure, intensity prescription, duration, frequency, supervision, and setting.

The most frequently used protocol was the 4 × 4 min interval model, performed at 85% to 95% HRpeak/HRmax with active recovery. This format was particularly common in cardiovascular and stroke rehabilitation studies. Alternative protocols included 30 s sprint intervals, 1 min intervals, and 6 s supramaximal bouts.

Exercise modalities included walking or running, cycle ergometry, resistance-based and multicomponent training, aquatic exercise, and other alternative formats. Most interventions were supervised, although some studies examined partially supervised or home-based approaches.

Intervention duration ranged from 4 weeks to 5 years, with weekly training frequencies varying between one and five sessions per week. Active recovery predominated, whereas passive recovery was more common in sprint-based protocols. Intensity was prescribed using HRpeak/HRmax, VO_2_peak workload-based measures, or ratings of perceived exertion (RPE).

Detailed characteristics of all HIIT and AIT interventions are presented in Table 3, while Figure 2 summarizes the distribution of studies across population groups and intervention formats.

### 3.5. Physiological and Cardiorespiratory Outcomes

Cardiorespiratory outcomes were among the most frequently assessed domains. Improvements in VO_2_peak and exercise tolerance were commonly reported within HIIT/AIT groups across apparently healthy, cardiovascular, metabolic, pulmonary, and neurological populations. However, the comparative findings varied according to the comparator condition. In trials comparing HIIT/AIT with MICT or another structured exercise intervention, some outcomes favored HIIT/AIT, whereas others showed comparable improvements or no statistically significant between-group differences. Accordingly, improvement from baseline was not interpreted as evidence of superiority over an active comparator. Several studies using 4 × 4 min protocols at approximately 85–95% HRpeak/HRmax reported increases in aerobic capacity, while additional adaptations included changes in workload tolerance and oxygen uptake efficiency slope (OUES).

Several studies also assessed vascular, autonomic, and hemodynamic outcomes. Reported adaptations included changes in arterial compliance, autonomic modulation, baroreflex sensitivity, left ventricular remodeling, and exercise-related hemodynamic responses. In heart failure populations, selected studies reported improvements in LVEF, OUES, and related cardiovascular measures.

In COPD and mixed cardiorespiratory populations, improvements in aerobic capacity and exercise tolerance were reported following HIIT/AIT, although the strength of comparative evidence varied according to study design and comparator. The single retained post-stroke study reported improvements in aerobic fitness and selected mobility-related outcomes; however, evidence from a single study does not establish a general effect in post-stroke populations.

Across the included reports, favorable within-group changes were more frequent than clear evidence of superiority over an active exercise comparator. Where HIIT/AIT was compared with MICT or another structured exercise intervention, findings ranged from greater improvements in selected outcomes to comparable or non-significant between-group effects. Accordingly, the direction of change alone was not considered evidence of comparative efficacy.

### 3.6. Metabolic Outcomes

Metabolic outcomes were assessed primarily in participants with type 2 diabetes, cardiometabolic dysfunction, obesity, and sedentary lifestyles. Reported outcomes included glycemic regulation, insulin sensitivity, lipid profile, body composition, inflammation, and oxidative stress.

Several studies involving T2DM or metabolic dysfunction reported within-group improvements in insulin sensitivity, glycemic control, and related cardiometabolic markers following HIIT/AIT. Søgaard et al. [49] reported improvements in insulin sensitivity together with skeletal-muscle markers related to glucose metabolism, while Marcotte-Chénard et al. [38] reported favorable glycemic outcomes following low-volume walking-based HIIT in older women with T2DM. Moro et al. [39] also reported reductions in basal insulin following resistance-oriented interval training. However, these findings should not be interpreted as consistent evidence of superiority over active comparators, and some were derived from non-randomized or methodologically limited designs.

Several studies also assessed body composition. Resistance-oriented or combined HIIT interventions reported reductions in fat mass and/or favorable changes in body composition [36,38]. Because some of these programs incorporated resistance or other exercise components in addition to HIIT, the observed adaptations cannot be attributed exclusively to the interval-training component.

Reductions in selected inflammatory or oxidative-stress markers were also reported in clinical populations [23,41,44]. Specifically, Alcazar et al. [41] reported reductions in protein carbonylation, while Fu et al. [44] reported decreases in selected cardiovascular and inflammatory biomarkers.

**Figure 3:**
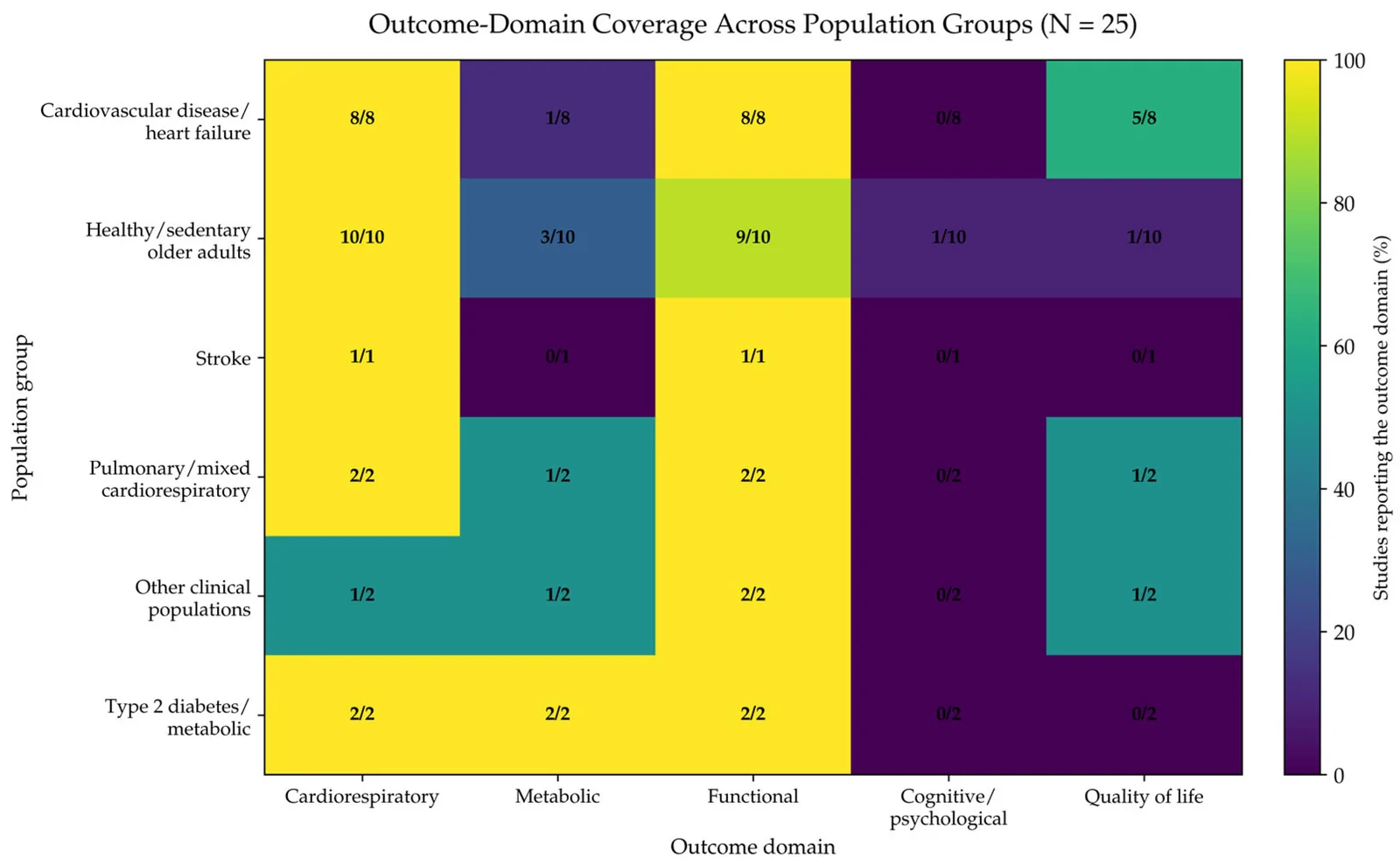
Outcome-domain coverage across population groups. Cells report the number of studies addressing each outcome domain relative to the total number of studies in the corresponding population group (n/N), while shading represents the percentage of studies. Outcome-domain coverage indicates that the outcome was reported and does not imply a favorable intervention effect.

Detailed metabolic outcomes are summarized in Table 4. Their distribution across population groups is shown in Figure 3.

**Table 4:** Main Outcomes of Included Studies.

Study	Main Outcomes Reported	Comparator/Context	Comparative Interpretation
Stensvold et al., (2020) [34]	VO_2_peak; physical function; QoL	MICT and control	Mixed comparative findings. Selected outcomes favored HIIT, but the findings do not establish overall superiority of HIIT.
Ellingsen et al., (2017) [35]	VO_2_peak; LV remodelling; exercise tolerance; QoL	MCT and RRE	Mixed findings. Improvements were reported, but consistent superiority over the active exercise comparators was not established.
Simonsson et al. (2023) [15]	VO_2_peak; global cognition; working memory	MIT	Mixed findings. No significant improvement in global cognition; selected between-group findings for working memory favored HIIT.
Frykholm et al. (2024) [36]	Neuromuscular performance; balance; strength	MIT	Improvements were reported in selected neuromuscular and functional outcomes. Comparative interpretation should be outcome-specific because this is a secondary report from the Umeå HIT Study.
Hwang et al. (2019) [24]	Exercise tolerance; metabolic regulation; functional capacity	MICT	Improvements were reported with HIIT; the current synthesis does not establish overall superiority over MICT.
Deka et al. (2022) [37]	CRF; body composition; ISWT; QoL	Usual care	Favorable outcomes were reported relative to usual care; however, the intervention combined HIIT and resistance training, so effects cannot be attributed exclusively to HIIT.
Marcotte-Chénard et al. (2021) [38]	VO_2_peak; glycaemic control; 6MWD; grip strength	MICT	Improvements were reported. Any claim of superiority over MICT should be restricted to outcomes showing a significant between-group effect.
Sculthorpe et al. (2017) [27]	Peak power; muscular power	Control	Selected improvements were reported relative to control; interpretation should remain outcome-specific.
Sian et al. (2022) [7]	VO_2_peak; blood pressure; lipid profile; exercise capacity	Laboratory HIIT and control	Physiological improvements were reported. Effects should be interpreted according to the specific comparison between home-based HIIT, laboratory HIIT, and control.
Jiménez-García et al. (2019) [14]	CRF; balance; gait/falls-related outcomes	MIIT and control	Selected functional improvements were reported; comparative superiority should be stated only for significant between-group findings.
Briggs et al. (2021) [23]	VO_2_peak; inflammatory markers; strength; endurance	AEX + RT	Selected benefits were reported in an active-comparator design; comparative effects should be interpreted outcome by outcome.
Moro et al. (2017) [39]	Strength; fat mass; functional strength	Traditional resistance training	Selected improvements were reported relative to resistance training; confidence is limited by methodological concerns.
Kim et al. (2017) [40]	Arterial compliance; functional capacity	MICT and control	Selected improvements were reported; between-group superiority should not be inferred from within-group changes alone.
García-Pinillos et al. (2019) [41]	CRF; mobility; strength	MICT	Improvements were reported; general superiority over MICT was not established in the current synthesis.
Alcazar et al. (2019) [42]	VO_2_peak; oxidative stress; muscular function	Control	Favorable outcomes were reported relative to control; however, the intervention also incorporated power training, limiting attribution specifically to HIIT.
Nilsson et al. (2008) [43]	Workload tolerance; 6MWT; QoL	Usual care	Improvements were reported relative to usual care; this should not be interpreted as evidence of superiority over an active exercise comparator.
Fu et al. (2016) [44]	LVEF; hemodynamics; functional capacity; QoL	General care	Improvements were reported relative to general care; comparative evidence is therefore different from an active-exercise comparison.
Fu et al. (2013) [45]	OUES; cerebral hemodynamics; exercise tolerance	MCT	Improvements were reported; comparative superiority over MCT should be restricted to outcomes with significant between-group differences.
Iellamo et al. (2013) [46]	VO_2_peak; hemodynamic responses; functional capacity	ACT	Improvements were reported in an active-comparator design; comparative interpretation should be based on between-group effects rather than within-group change alone.
Bressel et al. (2014) [47]	Balance; mobility; pain	Non-randomized control-period/pre–post design	Within-group/pre–post improvements. Causal inference is limited by the non-randomized design.
Mameletzi et al. (2011) [48]	Baroreflex sensitivity; aerobic tolerance	Control	Selected improvements were reported relative to control; interpretation should remain outcome-specific.
Søgaard et al. (2018) [49]	VO_2_max; insulin sensitivity; exercise performance	No adequate concurrent intervention comparator	Within-group improvements. Causal attribution to HIIT is limited by the absence of an adequate concurrent comparator.
Moholdt et al. (2012) [50]	VO_2_peak; functional recovery; QoL	Residential cardiac rehabilitation	Improvements were reported; the current synthesis does not establish overall superiority over residential rehabilitation.
Helgerud et al. (2010) [51]	VO_2_peak; workload capacity; QoL	Non-randomized/pre–post training; normoxia-related comparison	Pre–post improvements. Causal inference regarding the chronic training effect is limited by the absence of an adequate concurrent training comparator.
Kim et al. (2025) [52]	Aerobic fitness; balance; mobility	MICE	Selected aerobic and functional improvements were reported. Evidence remains limited to a single retained post-stroke study, and comparative interpretation should remain outcome-specific.

Notes. Main outcomes summarize the findings reported for the selected outcomes and should not be interpreted as evidence of overall intervention superiority. Comparative interpretation distinguishes within-group changes from between-group effects, comparisons with inactive/usual-care controls, and comparisons with active exercise interventions. Findings should be interpreted alongside the RoB 2 and ROBINS-I assessments presented in Table 5 and Table 6. AEX = aerobic exercise; ACT = aerobic continuous training; CRF = cardiorespiratory fitness; HIIT = high-intensity interval training; ISWT = Incremental Shuttle Walk Test; LV = left ventricular; MCT = moderate continuous training; MICT = moderate-intensity continuous training; MICE = moderate-intensity continuous exercise; MIT = moderate-intensity training; OUES = oxygen uptake efficiency slope; QoL = quality of life; RT = resistance training; 6MWD = six-minute walk distance.

**Table 5:** Risk of bias in randomized intervention studies assessed using the Cochrane RoB 2 tool.

Study	Result Assessed	Randomization Process	Period and Carryover Effects	Deviations from Intended Interventions	Missing Outcome Data	Measurement of the Outcome	Selection of the Reported Result	Overall Risk
Stensvold et al. (2020) [34]	All-cause mortality at 5 years	Low	N/A	Low	Low	Low	Low	Low
Ellingsen et al. (2017) [35]	LV end-diastolic diameter at 12 weeks	Some concerns	N/A	High	Low	Low	Low	High
Simonsson et al. (2023) [15]	VO_2peak_ and global cognition at 3 months	Low	N/A	Low	Low	Low	Low	Low
Frykholm et al. (2024) [36]	Muscular power and physical function at 3 months	Low	N/A	Low	Low	Low	Some concerns	Some concerns
Hwang et al. (2019) [24]	VO_2peak_ at 8 weeks	Low	N/A	Some concerns	High	Low	Some concerns	High
Deka et al. (2022) [37]	ISWT performance at 8 weeks	Low	N/A	Some concerns	Some concerns	Low	Some concerns	Some concerns
Marcotte-Chénard et al. (2021) [38]	6- minute walk distance at 12 weeks	Low	N/A	Low	Low	Low	Some concerns	Some concerns
Sculthorpe et al. (2017) [27]	Peak muscular power	Some concerns	N/A	High	Low	Low	Some concerns	Low
Sian et al. (2022) [7]	VO_2peak_ at 4 weeks	Some concerns	N/A	Low	Low	Some concerns	Low	Some concerns
Jiménez-García et al. (2019) [14]	Gait speed and quality of life at 12 weeks	Some concerns	N/A	Some concerns	Some concerns	High	Some concerns	High
Briggs et al. (2021) [23]	VO_2peak_ and functional performance	Some concerns	High	Some concerns	High	Low	Some concerns	High
Moro et al. (2017) [39]	Strength and body composition	Some concerns	N/A	Some concerns	High	Some concerns	Some concerns	High
Kim et al. (2017) [40]	Pulse-wave velocity at 8 weeks	Some concerns	N/A	Some concerns	High	Low	Some concerns	High
García-Pinillos et al. (2019) [41]	Mobility, strength and balance at 12 weeks	Some concerns	N/A	Some concerns	Low	Some concerns	Some concerns	Some concerns
Alcazar et al. (2019) [42]	VO_2peak_ and muscular function at 12 weeks	Some concerns	N/A	Some concerns	High	Some concerns	Some concerns	High
Nilsson et al. (2008) [43]	6MWT and quality of life at 4 and 12 months	Some concerns	N/A	Low	Low	Low	Some concerns	Some concerns
Fu et al. (2016) [44]	VO_2peak_ and hemodynamic response at 12 weeks	Some concerns	N/A	Some concerns	Some concerns	Some concerns	Some concerns	High
Fu et al. (2013) [45]	OUES and hemodynamic outcomes at 12 weeks	Some concerns	N/A	Some concerns	Some concerns	Some concerns	Some concerns	High
Iellamo et al. (2013) [46]	VO_2peak_ and hemodynamic	Some concerns	N/A	Some concerns	High	Some concerns	Some concerns	High
Mameletzi et al. (2011) [48]	Baroreflex sensitivity at 7 months	Some concerns	N/A	Some concerns	High	Some concerns	Some concerns	High
Moholdt et al. (2012) [50]	VO_2peak_ at 6 months	Low	N/A	Some concerns	Some concerns	Low	Low	Some concerns
Kim et al. (2025) [52]	VO_2peak_ and balance at 3 months	Low	N/A	Some concerns	Low	Low	Some concerns	Some concerns

Notes: RoB 2 = Cochrane Risk of Bias 2 tool; LV = left ventricular; VO_2_peak = peak oxygen uptake; ISWT = Incremental Shuttle Walk Test; 6MWT = six-minute walk test; OUES = oxygen uptake efficiency slope; N/A = not applicable. The domain concerning period and carryover effects was applied only to the crossover study by Briggs et al. [23]. RoB 2 judgments were made for the specific result indicated and should not necessarily be generalized to all outcomes reported in the same publication.

**Table 6:** Risk of bias in non-randomized intervention studies assessed using ROBINS-I.

Study	Result Assessed	Bias Due to Confounding	Bias in Selection of Participants	Bias in Classification of Interventions	Bias Due to Deviations from Intended Interventions	Bias Due to Missing Data	Bias in Measurement of Outcomes	Bias in Selection of the Reported Result	Overall Risk
Bressel et al. (2014) [47]	Pain, function, and mobility after 6 weeks	Serious	Moderate	Low	Moderate	Moderate	Serious	Moderate	Serious
Søgaard et al. (2018) [49]	Insulin sensitivity after 6 weeks	Critical	Serious	Low	Moderate	Moderate	Low	Serious	Critical
Helgerud et al. (2010) [51]	VO_2peak_ after 24 training sessions	Critical	Serious	Moderate	Moderate	Moderate	Low	Serious	Critical

Notes: ROBINS-I = Risk Of Bias In Non-randomized Studies of Interventions. Judgments were categorized as low, moderate, serious, or critical risk of bias. A serious judgment indicates important methodological problems that substantially limit confidence in the result. A critical judgment indicates that the study cannot provide a reliable causal estimate for the intervention effect under assessment. The application of ROBINS-I to the single-group studies by Søgaard et al. and Helgerud et al. was limited by the absence of an adequate concurrent comparator.

### 3.7. Functional Outcomes

Functional outcomes included walking capacity, muscular strength, balance, mobility, and related measures of physical performance. Improvements from baseline were reported across several cardiovascular, pulmonary, metabolic, and apparently healthy populations, including changes in six-minute walk distance (6MWD), Incremental Shuttle Walk Test (ISWT) performance, muscular strength, peak power, and balance. However, the magnitude and comparative significance of these changes varied across studies, and within-group improvement did not consistently correspond to a significant advantage over an active comparator.

In the retained post-stroke study, skating-based HIIT was associated with improvements in aerobic fitness, balance, and mobility. Alternative interval modalities, including resistance-oriented, multicomponent, and aquatic programmes, also reported selected functional improvements. Because some interventions incorporated additional resistance or power-training components, attribution of these functional effects specifically to HIIT should be made cautiously.

Detailed functional outcomes are summarised in Table 4 and Figure 3.

### 3.8. Cognitive Outcomes

Cognitive outcomes were investigated in only a small proportion of the retained evidence base. In the Umeå HIT Study, three months of low-volume supramaximal HIIT did not significantly improve global cognitive function in sedentary older adults. However, between-group differences in working memory favoured the HIIT condition.

Accordingly, evidence regarding cognitive effects remains limited and does not currently support broad conclusions regarding the effects of HIIT on cognitive function in older adults.

### 3.9. Quality of Life and Psychological Outcomes

Health-related quality of life (HRQoL) was assessed in a subset of the retained studies, particularly in cardiovascular and pulmonary populations. Several studies reported improvements in HRQoL or perceived physical functioning, although these findings included both within-group changes and selected comparative effects and were not consistent across interventions or populations. In the Generation 100 study, long-term HIIT showed favorable maintenance of physical and mental HRQoL components relative to the physical activity recommendation group. Conversely, the Umeå HIT Study reported no significant improvement in global quality of life following low-volume supramaximal HIIT.

Specific psychological outcomes were assessed infrequently in the retained evidence base, precluding firm conclusions regarding anxiety, depression, or other psychological domains.

Patient preference was not systematically assessed across the included studies. Adherence, tolerability, acceptability, and exercise-related perceptions were reported more frequently, but these outcomes should not be interpreted as direct measures of participant preference. Accordingly, evidence regarding patient preference remains limited.

Detailed quality-of-life and patient-reported outcomes are summarized in Table 4 and Figure 3.

### 3.10. Safety, Feasibility, and Adherence

Feasibility and adherence were generally favorable in studies reporting these outcomes, although safety reporting was inconsistent across the evidence base. Most reported interventions achieved moderate-to-high adherence, particularly under supervised conditions. Sian et al. reported complete adherence in both laboratory- and home-based HIIT groups, while the Umeå HIT Study reported approximately 88% attendance. Moro et al. also reported higher retention with interval resistance training than conventional resistance training (78% vs. 53%).

Feasibility was demonstrated in selected clinical populations, including severely deconditioned participants with type 2 diabetes mellitus. However, the predominantly supervised nature of many interventions should be considered when interpreting generalizability to routine or unsupervised settings.

Adverse-event reporting varied substantially across studies. Of the events described, many were musculoskeletal and transient. In the Umeå HIT Study, musculoskeletal complaints accounted for approximately 70% of documented adverse events, with no major cardiovascular complications reported. In the Generation 100 study, no cardiovascular adverse events occurred during supervised exercise sessions, although three fractures associated with outdoor slipping incidents occurred during unsupervised training. In SMARTEX-HF, serious adverse events were numerically more frequent in the HIIT group, but between-group differences were not statistically significant.

Because adverse events were not uniformly defined or systematically reported, the available evidence supports the feasibility of HIIT/AIT in selected and frequently supervised settings more clearly than it establishes comparative safety across older or clinically complex populations.

### 3.11. Risk of Bias

The risk of bias was assessed separately for each study design. Of the 22 randomized reports evaluated using RoB 2, two results were judged at low risk of bias, eight raised some concerns, and twelve were judged at high risk of bias. The most frequent concerns involved deviations from the intended interventions, incomplete outcome data, and insufficient information to exclude selective reporting. Lack of participant or exercise-professional blinding was not considered, by itself, sufficient to classify an exercise trial as being at high risk of bias.

Three non-randomized studies were evaluated using ROBINS-I. One was judged at serious risk of bias and two at critical risk of bias. The principal concerns involved uncontrolled confounding, absence of an appropriate concurrent comparator, participant selection, and limitations in attributing observed changes specifically to the HIIT/AIT intervention.

These judgments reduced confidence in the causal interpretation of favorable within-group changes. Greater interpretative weight was therefore assigned to randomized between-group results judged at low risk of bias. Findings from high-risk randomized results and from non-randomized studies were interpreted as supportive or hypothesis-generating evidence rather than as definitive evidence of efficacy, superiority, or safety.

Detailed domain-level risk-of-bias judgments are presented in Table 5 and Table 6.

Overall, the risk-of-bias assessment indicated that the evidence base was methodologically limited. Only two randomized results were judged at low risk of bias. Most randomized results raised some concerns or were judged at high risk, while all non-randomized studies presented serious or critical risk-of-bias concerns. These findings were considered when interpreting the direction and strength of the evidence, particularly when studies reported favourable within-group changes without a significant between-group effect.

## 4. Discussion

The present systematic review synthesized evidence on HIIT/AIT in older adults across apparently healthy and selected clinical populations using a uniform age criterion and design-specific risk-of-bias assessment. The principal pattern was a comparatively stronger and more consistent evidence base for cardiorespiratory fitness and selected functional outcomes, whereas evidence for other health domains was less extensive or less consistent. Beyond summarizing intervention effects, the present review extends previous syntheses by examining a broader range of clinical and patient-relevant outcomes, differentiating findings according to comparator type and study design, and considering how heterogeneity in HIIT/AIT prescription influences interpretation. Accordingly, the findings support HIIT/AIT as a potentially useful exercise option in selected older populations, while also demonstrating that its effects cannot be generalized across populations, outcomes, or prescription models.

The risk-of-bias assessment materially qualifies the generally favorable direction of the findings. Only two of the 22 randomized results were judged at low risk of bias, while eight raised some concerns and twelve were judged at high risk. The three non-randomized studies presented serious or critical risk-of-bias concerns. These judgments do not necessarily invalidate the reported findings, but they reduce confidence in causal attribution and in the magnitude of the observed effects. Consequently, favorable within-group changes should not be interpreted as robust evidence of comparative efficacy, and greater interpretative weight should be given to low-risk randomized results demonstrating between-group effects.

Cardiorespiratory fitness represented the outcome domain with the greatest convergence across the included evidence. Improvements in VO_2_peak or related indices of aerobic capacity were reported across apparently healthy and selected clinical populations, although the magnitude of change and comparative advantage over MICT varied between studies. Several methodological and prescription factors may contribute to this variability. Protocols differed markedly in exercise intensity, interval duration, recovery structure, total training volume, intervention duration, and weekly frequency, producing substantially different training stimuli despite their common classification as HIIT/AIT. Baseline fitness may also influence the magnitude of adaptation, because participants with lower initial cardiorespiratory capacity may have greater potential for improvement than relatively fit older adults. Supervised protocols may additionally facilitate closer attainment of prescribed intensities and better intervention fidelity than less supervised approaches. Finally, comparisons with MICT require particular caution when exercise volume, session duration, or energy expenditure are not equivalent between groups, because observed differences cannot necessarily be attributed to intensity alone. These factors may help explain why some studies favored HIIT/AIT whereas others reported broadly comparable adaptations, supporting HIIT/AIT as a viable strategy for improving cardiorespiratory fitness without establishing its general superiority over MICT.

The applicability and magnitude of HIIT/AIT responses are also likely to depend on the population studied. Apparently healthy or sedentary older adults may retain sufficient physiological reserve to tolerate relatively high workloads and progress exercise intensity with fewer disease-related constraints. In contrast, cardiovascular populations may be limited by impaired cardiac output, chronotropic response, exercise intolerance, or concurrent pharmacological treatment, which can influence both achievable exercise intensity and physiological adaptation. Metabolic disorders may additionally involve insulin resistance, obesity, and skeletal-muscle dysfunction, whereas pulmonary disease can restrict training through ventilatory limitation and dyspnea. Neurological conditions may introduce further constraints related to balance, motor control, gait impairment, and mobility. Medication use, multimorbidity, baseline functional capacity, and disease severity may therefore modify both the tolerability and responsiveness to HIIT/AIT. These considerations help explain why findings observed in relatively healthy older adults cannot be assumed to translate directly to cardiovascular, metabolic, pulmonary, neurological, frail, or multimorbid populations.

Feasibility and adherence were generally favourable in studies reporting these outcomes, particularly under structured or supervised conditions, but these observations should not be equated with comparative safety. Adverse events were inconsistently defined and monitored, and several studies involved relatively small or clinically selected samples. In SMARTEX-HF, for example, serious adverse events were numerically more frequent in the HIIT group, although between-group differences were not statistically significant. Moreover, the considerable diversity of intervention models—including differences in intensity, interval structure, frequency, modality, supervision, and setting—precludes identification of a single optimal HIIT/AIT prescription. This diversity demonstrates that interval-based exercise can be adapted to different contexts, but translation to routine clinical or unsupervised practice should remain conditional on individual clinical assessment, appropriate progression, and supervision where indicated.

Functional outcomes showed generally favorable patterns, including improvements in walking capacity, balance, mobility, muscular strength, and power. These outcomes are clinically relevant because they represent physiological and motor capacities that contribute to independent mobility and activities of daily living. However, improvements in performance-based tests should not automatically be interpreted as equivalent improvements in real-world independence. Measures such as the 6MWT, ISWT, balance tests, or isolated strength and power assessments primarily quantify specific components of physical capacity and may not capture complex activities such as transferring, stair negotiation, domestic tasks, community mobility, or sustained independent living. Moreover, relatively few studies assessed disability, activities of daily living, or other directly patient-centered functional endpoints. The current evidence therefore supports improvement in selected components of functional capacity more clearly than it demonstrates improved independence in everyday life. This distinction is particularly important because the evidence base remained concentrated in apparently healthy or cardiovascular populations, with substantially less evidence in neurological, pulmonary, frail, and multimorbid older adults.

Metabolic outcomes provided additional evidence of potential benefit, particularly among individuals with type 2 diabetes mellitus, metabolic dysfunction, or sedentary lifestyles. Selected studies reported improvements in insulin sensitivity, glycemic regulation, lipid profiles, body composition, and inflammatory or oxidative-stress markers. These findings are compatible with the established metabolic responsiveness to vigorous aerobic and resistance exercise; however, metabolic endpoints were less consistently assessed than cardiorespiratory fitness and were measured using diverse techniques. Furthermore, some interventions incorporated resistance training or other exercise components in addition to HIIT. The present evidence therefore suggests potential metabolic benefits but does not permit attribution of all observed adaptations specifically to HIIT or identification of an optimal interval-training dose for metabolic health.

In contrast, evidence regarding cognitive and psychological outcomes remains limited. Within the retained evidence base, the Umeå HIT trial did not demonstrate a significant improvement in global cognitive function following low-volume supramaximal HIIT, although selected between-group differences favored HIIT for working memory. This distinction is important because improvements in individual cognitive domains should not be interpreted as evidence of a general cognitive effect. Likewise, psychological outcomes were assessed relatively infrequently after application of the revised eligibility criteria. Several studies that contributed anxiety, depression, or neurocognitive findings to the original synthesis no longer met the uniform age criterion and were therefore excluded from the revised review. Current evidence is consequently insufficient to conclude that HIIT produces consistent improvements in global cognition, anxiety, depression, or broader psychological well-being in older adults. These outcomes warrant further investigation using standardized instruments and adequately powered trials.

Health-related quality of life showed similarly heterogeneous findings. Improvements were reported in selected cardiovascular and pulmonary studies, and long-term evidence from the Generation 100 trial suggested favorable maintenance of some health-related quality-of-life components. Conversely, other interventions did not demonstrate significant changes in global quality of life despite improvements in objective physiological outcomes. This divergence highlights an important distinction between physiological adaptation and patient-perceived benefit. In addition, participant preference was rarely assessed directly. Adherence, retention, exercise enjoyment, tolerability, and acceptability may provide useful information regarding intervention uptake, but they should not be considered interchangeable with preference. Consequently, the present review cannot determine whether older adults generally prefer HIIT to other exercise modalities.

Some reports originated from the same parent trial and were therefore treated as related publications rather than independent replications; consequently, multiple reports from a single cohort should not be interpreted as increasing the amount of independent evidence supporting an intervention effect.

The principal contribution of the present review is to provide an updated and clinically differentiated synthesis of HIIT/AIT evidence across a broader range of outcomes and older populations than has typically been considered in previous reviews. Rather than supporting universal superiority of HIIT/AIT, the synthesis identifies where evidence is comparatively stronger—particularly for cardiorespiratory fitness and selected components of physical function—and where substantial uncertainty persists, including cognitive, psychological, preference, safety, and less frequently studied clinical outcomes. Clinically, HIIT/AIT may therefore represent an alternative exercise strategy for apparently healthy or sedentary older adults and selected clinically stable cardiovascular populations when appropriately prescribed and monitored. Application to metabolic, pulmonary, neurological, frail, or multimorbid populations should be more cautious because the available evidence is smaller, more heterogeneous, and methodologically less robust. Exercise prescription should consequently remain individualized according to baseline functional capacity, clinical stability, comorbidities, medication use, previous exercise experience, treatment goals, and the level of supervision available.

Future research should prioritize adequately powered randomized trials in populations that remain underrepresented, particularly frail and multimorbid older adults. Trials should use reproducible HIIT/AIT protocols and compare HIIT with MICT under appropriately matched conditions of exercise volume or energy expenditure to better isolate the effect of training intensity. Standardized and prospective adverse-event surveillance is needed to establish comparative safety, while participant preference should be assessed directly rather than inferred from adherence, enjoyment, or retention. Greater emphasis should also be placed on clinically meaningful functional outcomes, including activities of daily living, disability, mobility in real-world settings, and maintenance of independence. Longer follow-up is required to determine whether improvements in physiological or laboratory-based functional measures translate into sustained patient-relevant benefits.

## 5. Limitations, Practical Implications and Future Directions

Several limitations should be considered when interpreting this review. First, the review was retrospectively registered, and study selection was conducted by one primary reviewer rather than independently in duplicate. Second, eligibility of mixed-age samples was based on the reported study-level mean or median age when age-stratified data were unavailable; consequently, some included samples contained participants younger than 60 years. Third, substantial clinical and methodological heterogeneity across populations, intervention protocols, comparators, and outcome measures precluded quantitative pooling and limits direct comparison between studies. This issue is particularly relevant for interventions combining HIIT/AIT with resistance or power training, for which observed effects cannot be attributed exclusively to the interval-training component. Confidence in the findings is further limited by the predominance of risk-of-bias concerns, the absence of a formal certainty-of-evidence assessment, and inconsistent adverse-event reporting. Finally, multiple reports from the same parent trial, restriction to peer-reviewed English-language publications, and the predominance of selected and supervised populations may limit the independence and generalizability of the evidence.

From a practical perspective, the available evidence supports HIIT/AIT as a potential alternative exercise strategy for selected older adults, particularly for improving cardiorespiratory fitness and selected components of physical function. However, the findings do not establish general superiority over MICT, comparative safety across populations, or a single optimal prescription. Implementation should therefore remain individualized according to clinical stability, functional capacity, comorbidities, previous exercise experience, and the availability of appropriate supervision.

Future research should prioritize adequately powered randomized trials involving frail and multimorbid older adults, direct assessment of participant preference, standardized prospective adverse-event surveillance, and clinically meaningful functional outcomes such as activities of daily living and maintenance of independence. Comparisons between HIIT/AIT and MICT should, where possible, use equivalent exercise volume or energy expenditure to better isolate the influence of training intensity. Longer follow-up is also required to establish whether physiological and performance-based improvements translate into sustained patient-relevant benefits.

## 6. Conclusions

This systematic review suggests that HIIT/AIT may improve cardiorespiratory fitness, particularly VO_2_peak and exercise tolerance, together with selected measures of physical and functional performance in older adults. These findings were most frequently reported in apparently healthy or sedentary older adults and in selected cardiovascular populations. Potential metabolic benefits were also identified, whereas evidence concerning cognitive, psychological, health-related quality-of-life, and long-term clinical outcomes was less extensive and less consistent.

When HIIT/AIT was compared with MICT or another active exercise intervention, results ranged from greater improvements in selected outcomes to comparable or non-significant between-group effects. The available evidence therefore supports HIIT/AIT as a potentially viable alternative exercise strategy but does not establish its general superiority. The substantial heterogeneity of the populations, intervention protocols, comparators, and outcome measures also prevents identification of a single optimal HIIT/AIT prescription.

Confidence in these conclusions is limited by the predominance of some concerns or high risk of bias among randomized results and by serious or critical risk of bias among the non-randomized evidence. In addition, certainty of the evidence was not formally assessed, and adverse events were inconsistently defined and reported. The evidence supports feasibility in selected and frequently supervised settings more clearly than it establishes broad comparative safety.

Accordingly, implementation of HIIT/AIT in older or clinically complex adults should remain conditional upon individual clinical and functional assessment, appropriate progression and monitoring, and supervision when indicated. Further adequately powered and methodologically rigorous comparative trials are needed before firm recommendations can be made regarding superiority, long-term safety, optimal prescription, and effectiveness in frail, multimorbid, neurological, pulmonary, and unsupervised populations.

Take-Home Messages

- HIIT/AIT may improve cardiorespiratory fitness and selected functional outcomes, particularly in apparently healthy or sedentary older adults and selected cardiovascular populations.
- Most randomized results raised some concerns or were judged at high risk of bias, while the non-randomized evidence presented serious or critical risk-of-bias concerns.
- The strongest and most consistent evidence relates to VO_2_peak, exercise tolerance, walking capacity, and selected measures of muscular and functional performance.
- Metabolic, cognitive, psychological, and quality-of-life outcomes show potentially favorable but less consistent findings.
- Considerable heterogeneity exists in HIIT/AIT modality, interval structure, intensity prescription, intervention duration, and participant characteristics, limiting direct comparison across studies.
- Feasibility and adherence were generally favorable in selected and frequently supervised settings; however, adverse events were not systematically reported, and comparative safety remains insufficiently established.
- Patient preference was rarely assessed directly; therefore, adherence, tolerability, enjoyment, and acceptability should not be interpreted as equivalent to preference.
- Findings from high-risk and non-randomized evidence should be considered supportive or hypothesis-generating rather than definitive evidence of efficacy, superiority, or safety.
- Future studies should use more standardized intervention protocols, outcome measures, adverse-event reporting, and longer follow-up to strengthen exercise-prescription recommendations.

## Figures and Tables

**Table 1:** Characteristics of the included studies.

Study	Population	n	Age (Years)	Design	HIIT/AIT Protocol	Comparator	Duration	Follow-Up	Main Outcomes
Stensvold et al., (2020) [34]	Healthy older adults	1567	70–77	RCT	2×/week; 4 × 4 min at ~90% peak HR	MICT and Control	5 years	5 years	Mortality, VO_2_peak, QoL
Ellingsen et al., (2017) [35]	HFrEF	231	60, median (IQR 53–70)	Multicenter RCT	4 × 4 min at 90–95% peak HR	MCT and RRE	12 weeks	52 weeks	LV remodeling, VO_2_peak
Simonsson et al., (2023) [15]	Sedentary older adults	68	66–79	RCT	2×/week; 10 × 6 s supramaximal intervals	MIT (69% MAP)	3 months	–	Global cognition, VO_2_peak
Frykholm et al., (2024) [36]	Sedentary older adults	68	66–79	RCT	2×/week; 10 × 6 s supramaximal intervals	MIT (69% MAP)	3 months	–	Muscle strength, balance
Hwang et al. (2019) [24]	Type 2 diabetes mellitus	58	63 ± 1 (range 46–78)	RCT	4×/week; 4 × 4 min at 90% peak HR	MICT	8 weeks	–	VO_2_peak, exercise tolerance
Deka et al. (2022) [37]	Coronary artery disease	90	~69	RCT	1×/week; 10 × 1 min at 85–90% MHR + RT	Usual care	8 weeks	–	Body composition, ISWT, QoL
Marcotte-Chénard et al. (2021) [38]	Women with T2DM	29	~67.7	RCT	Low-volume walking HIIT	MICT	12 weeks	–	6MWD, VO_2_peak, grip strength
Sculthorpe et al. (2017) [27]	Sedentary men	33	~62	RCT	1 session every 5 days; 6 × 30 s sprints	Control	6 weeks	–	Peak power output
Sian et al. (2022) [7]	Healthy older adults	30	~71	RCT	3×/week; 5 × 1 min > 85% HRmax	HIIT Lab and CON	4 weeks	–	VO_2_peak, blood pressure, lipids
Jiménez-García et al. (2019) [14]	Healthy older adults	73	~68	RCT	2×/week; 4 × 4 min at 90–95% HRmax (TRX)	MIIT and CON	12 weeks	–	Falls risk, gait, balance
Briggs et al. (2021) [23]	HIV (50+ years)	26	61.5 ± 6.7	RCT	3×/week; 4 × 4 min at 90–95% HRmax + RT	AEX + RT	4 months	–	VO_2_peak, strength, endurance
Moro et al. (2017) [39]	Healthy older adults	35	60–80	RCT	HIIRT (resistance-based HIIT)	TRT	2 months	–	Strength, body composition, lipids
Kim et al. (2017) [40]	Sedentary older adults	49	~65	RCT	4×/week; 4 × 4 min at 90% peak HR	MICT and CON	8 weeks	–	Arterial stiffness, VO_2_peak
García-Pinillos et al. (2019) [41]	Active older adults	90	~72.8	RCT	Concurrent HIIT	MICT	12 weeks	–	Mobility, balance, strength
Alcazar et al. (2019) [42]	COPD (65+)	29	≥65	RCT	10 × 30 s at 80% Wpeak + power training	Control	12 weeks	–	Oxidative stress, VO_2_peak
Nilsson et al. (2008) [43]	CHF (NYHA II–III)	80	68.8	RCT	Ullevaal interval model	Usual care	4 months	12 months	6MWT, workload, QoL
Fu et al. (2016) [44]	HFpEF/HFrEF	120	66–73	RCT	3×/week; 3 min intervals at 80% VO_2_peak	General care	12 weeks	–	LVEF, VO_2_peak, hemodynamics
Fu et al. (2013) [45]	Heart failure	45	66–68	RCT	3×/week; 3 min intervals at 80% VO_2_peak	MCT	12 weeks	–	OUES, cerebral hemodynamics
Iellamo et al. (2013) [46]	Heart failure	20	62.2	RCT	Dose-matched 4 × 4 min HIIT	ACT	12 weeks	–	VO_2_peak, hemodynamics
Bressel et al. (2014) [47]	Osteoarthritis	18	64.5	Clinical trial	Aquatic HIIT	Control period	6 weeks	–	Joint pain, function, balance
Mameletzi et al. (2011) [48]	Coronary artery disease	25	69–71	RCT	Structured interval training	Control	7 months	7 months	Baroreflex sensitivity
Søgaard et al. (2018) [49]	Sedentary older adults	22	~63	RCT	3×/week; 5 × 1 min cycling intervals	Gender comparison	6 weeks	–	Insulin sensitivity, VO_2_max
Moholdt et al. (2012) [50]	Post-CABG	30	~63	RCT	Home-based 4 × 4 min HIIT	Residential rehabilitation	4 weeks	6 months	VO_2_peak, HR recovery, QoL
Helgerud et al. (2010) [51]	COPD and CAD	14	61–64	RCT	4 × 4 min at 85–95% HRpeak	Normoxia	10 weeks	–	VO_2_peak, workload, QoL
Kim et al. (2025) [52]	Older post-stroke adults	36	70.7	RCT	Skating exercise-based HIIT	MICE	3 months	–	VO_2_peak, balance, SMI

Notes. HIIT = high-intensity interval training; AIT = aerobic interval training; MICT = moderate-intensity continuous training; CHF = chronic heart failure; HFrEF = heart failure with reduced ejection fraction; HFpEF = heart failure with preserved ejection fraction; COPD = chronic obstructive pulmonary disease; CAD = coronary artery disease; CABG = coronary artery bypass graft; QoL = quality of life; VO_2_peak = peak oxygen uptake; 6MWT = six-minute walk test; BBS = Berg Balance Scale; HRV = heart rate variability; ISWT = Incremental Shuttle Walk Test; SMI = skeletal muscle index. The FITR Heart Study protocol was excluded from the final qualitative synthesis due to the absence of longitudinal intervention outcomes. Age values are presented using the summary statistic reported in the original study. For mixed-age samples, eligibility was based on a reported mean or median age ≥ 60 years when age-stratified data were unavailable; no minimum proportion of participants aged ≥60 years was required. Studies with a mean or median age < 60 years were retained only when data for participants aged ≥60 years were separately extractable.

**Table 2:** Participant and Clinical Characteristics of Included Studies.

Study	Sex Distribution	Clinical Condition/Population	Baseline Functional Assessment (Tool/Value)	Baseline VO_2_peak/CRF	Relevant Comorbidities	Clinical Notes
Stensvold et al., (2020) [34]	Mixed	Healthy older adults	NR	Reduced age-related CRF	Hypertension, overweight	Community-dwelling older adults
Ellingsen et al., (2017) [35]	Predominantly male	HFrEF	NYHA II–III	Reduced VO_2_peak	CHF, hypertension	Multicenter cardiac rehabilitation cohort
Simonsson et al. (2023) [15]	Mixed	Sedentary older adults	Functional independence preserved	Reduced CRF	Sedentary lifestyle	Non-exercising older adults
Frykholm et al. (2024) [36]	Mixed	Sedentary older adults	Balance and strength deficits	Reduced CRF	Sedentary lifestyle	Secondary analysis of Umeå HIT Study
Hwang et al. (2019) [24]	Mixed	Type 2 diabetes mellitus	Exercise intolerance	Reduced VO_2_peak	T2DM, obesity	Older adults with metabolic dysfunction
Deka et al. (2022) [37]	Predominantly male	Coronary artery disease	ISWT impairment	Reduced CRF	CAD, hypertension	Combined HIIT + resistance intervention
Marcotte-Chénard et al. (2021) [38]	Female	Type 2 diabetes mellitus	Reduced 6MWD	Reduced CRF	T2DM, overweight	Older women only
Sculthorpe et al. (2017) [27]	Male	Sedentary older adults	Reduced muscle power	Reduced CRF	Sedentary lifestyle	Aging-associated sarcopenic decline
Sian et al. (2022) [7]	Mixed	Healthy older adults	Preserved mobility	Moderately reduced CRF	Sedentary behavior	Home-based HIIT feasibility
Jiménez-García et al. (2019) [14]	Mixed	Healthy older adults	Increased falls risk	NR	Balance impairment	Suspension training intervention
Briggs et al. (2021) [23]	Mixed	HIV-associated aging	Reduced endurance	Reduced VO_2_peak	HIV, inflammation	Accelerated biological aging
Moro et al. (2017) [39]	Mixed	Healthy older adults	Preserved mobility	NR	Age-related adiposity	Resistance-oriented HIIT
Kim et al. (2017) [40]	Mixed	Sedentary older adults	Functional independence	Reduced CRF	Vascular stiffness	Arterial function focus
García-Pinillos et al. (2019) [41]	Mixed	Active older adults	Preserved mobility	Moderate CRF	Aging-related decline	Concurrent training model
Alcazar et al. (2019) [42]	Mixed	COPD	Functional impairment	Reduced VO_2_peak	COPD, muscle dysfunction	Oxidative stress analysis
Nilsson et al. (2008) [43]	Mixed	CHF	Reduced 6MWT	Reduced CRF	CHF	Ullevaal rehabilitation model
Fu et al. (2016) [44]	Mixed	HFpEF/HFrEF	Reduced exercise tolerance	Reduced VO_2_peak	HF	Hemodynamic assessment
Fu et al. (2013) [45]	Mixed	Heart failure	Reduced functional capacity	Reduced VO_2_peak	HF	Cerebral hemodynamics focus
Iellamo et al. (2013) [46]	Mixed	Heart failure	Reduced exercise tolerance	Reduced CRF	HF	Dose-matched training comparison
Bressel et al. (2014) [47]	Mixed	Osteoarthritis	Mobility impairment	NR	OA-related pain	Aquatic exercise intervention
Mameletzi et al. (2011) [48]	Mixed	Coronary artery disease	Reduced aerobic tolerance	Reduced CRF	CAD	Autonomic function analysis
Søgaard et al. (2018) [49]	Mixed	Sedentary older adults	Preserved mobility	Reduced VO_2_max	Insulin resistance	Sex-based physiological responses
Moholdt et al. (2012) [50]	Predominantly male	Post-CABG	Reduced exercise tolerance	Reduced VO_2_peak	CAD	Home-based rehabilitation
Helgerud et al. (2010) [51]	Mixed	COPD and CAD	Reduced functional capacity	Reduced VO_2_peak	COPD, CAD	Hyperoxia-based HIIT
Kim et al. (2025) [52]	Mixed	Older post-stroke adults	Balance and mobility deficits	Reduced VO_2_peak	Stroke sequelae	Skating-based HIIT intervention

Notes. CRF = cardiorespiratory fitness; CHF = chronic heart failure; HF = heart failure; HFrEF = heart failure with reduced ejection fraction; HFpEF = heart failure with preserved ejection fraction; COPD = chronic obstructive pulmonary disease; CAD = coronary artery disease; CABG = coronary artery bypass graft; 6MWT = six-minute walk test; 6MWD = six-minute walk distance; ISWT = Incremental Shuttle Walk Test; BBS = Berg Balance Scale; QoL = quality of life; NR = not reported.

**Table 3:** Characteristics of HIIT/AIT Protocols Implemented Across Included Studies.

Study	Exercise Modality	Interval Structure	Work:Rest Ratio	Intensity Prescription	Recovery Type	Session Duration	Weekly Frequency	Supervision	Setting
Stensvoldet al., (2020) [34]	Walking/running	4 × 4 min	1:0.75	85–95% HRpeak	Active	~40 min	2×/week	Supervised	Community/laboratory
Ellingsen et al., (2017) [35]	Treadmill/cycle	4 × 4 min	1:0.75	90–95% HRmax	Active	~38–40 min	3×/week	Supervised	Clinical rehabilitation
Simonsson et al. (2023) [15]	Cycle ergometer	10 × 6 s supramaximal	1:9	Supramaximal/Wmax	Passive	~20 min	2×/week	Supervised	Laboratory
Frykholm et al. (2024) [36]	Cycle ergometer	10 × 6 s supramaximal	1:9	Supramaximal/Wmax	Passive	~20 min	2×/week	Supervised	Laboratory
Hwang et al. (2019) [24]	Total-body ergometer	4 × 4 min	1:0.75	85–95% HRpeak	Active	~40 min	4×/week	Supervised	Clinical/laboratory
Deka et al. (2022) [37]	Cycle ergometer + RT	10 × 1 min	1:1	85–90% MHR	Active	~35–40 min	1×/week	Supervised	Rehabilitation center
Marcotte-Chénard et al. (2021) [38]	Walking	Low-volume intervals	NR	Vigorous intensity	Active	~20–25 min	3×/week	Supervised	Community
Sculthorpe et al. (2017) [27]	Cycle sprinting	6 × 30 s sprints	1:4	Maximal effort	Passive	~20 min	Every 5 days	Supervised	Laboratory
Sian et al. (2022) [7]	Walking/running	5 × 1 min	1:1	>85% HRmax	Active	~25–30 min	3×/week	Home/lab supervised	Home/laboratory
Jiménez-García et al. (2019) [14]	TRX suspension training	4 × 4 min	1:1	90–95% HRmax	Active	~40 min	2×/week	Supervised	Gymnasium
Briggs et al. (2021) [23]	Treadmill + RT	4 × 4 min	1:0.75	90–95% HRmax	Active	~45 min	3×/week	Supervised	Clinical/laboratory
Moro et al. (2017) [39]	Resistance training	HIIRT	Variable	High-load resistance	Passive	~40 min	2×/week	Supervised	Gymnasium
Kim et al. (2017) [40]	Treadmill/cycle	4 × 4 min	1:0.75	90% HRpeak	Active	~40 min	4×/week	Supervised	Laboratory
García-Pinillos et al. (2019) [41]	Concurrent training	Variable intervals	Variable	Vigorous intensity	Active	~45–60 min	2×/week	Supervised	Exercise facility
Alcazar et al. (2019) [42]	Cycle ergometer + power training	10 × 30 s	1:2	80% Wpeak	Active	~35–40 min	2–3×/week	Supervised	Rehabilitation
Nilsson et al. (2008) [43]	Group aerobic circuit	Ullevaal intervals	Variable	Borg scale 15–17	Active	~50 min	2×/week	Supervised	Cardiac rehabilitation
Fu et al. (2016) [44]	Cycle ergometer	3 min intervals	1:1	80% VO_2_peak	Active	~30–40 min	3×/week	Supervised	Hospital/laboratory
Fu et al. (2013) [45]	Cycle ergometer	3 min intervals	1:1	80% VO_2_peak	Active	~30–40 min	3×/week	Supervised	Hospital/laboratory
Iellamo et al. (2013) [46]	Cycle ergometer	4 × 4 min	1:0.75	Dose-matched HIIT	Active	~40 min	3×/week	Supervised	Hospital
Bressel et al. (2014) [47]	Aquatic treadmill	Aquatic intervals	Variable	Moderate-to-high intensity	Active	~30–45 min	3×/week	Supervised	Aquatic facility
Mameletzi et al. (2011) [48]	Cycle ergometer	Structured intervals	Variable	Vigorous intensity	Active	~40 min	3×/week	Supervised	Rehabilitation
Søgaard et al. (2018) [49]	Cycle ergometer	5 × 1 min	1:1	Near-maximal intensity	Active	~25 min	3×/week	Supervised	Laboratory
Moholdt et al. (2012) [50]	Home-based walking/running	4 × 4 min	1:0.75	85–95% HRpeak	Active	~40 min	2×/week	Partially supervised	Home/community
Helgerud et al. (2010) [51]	Treadmill walking	4 × 4 min	1:0.75	85–95% HRpeak	Active	~40 min	3×/week	Supervised	Clinical rehabilitation
Kim et al. (2025) [52]	Skating exercise	Skating intervals	Variable	Vigorous intensity	Active	~40 min	3×/week	Supervised	Rehabilitation/gym

Notes. HIIT = high-intensity interval training; AIT = aerobic interval training; HIIRT = high-intensity interval resistance training; RT = resistance training; HRmax = maximum heart rate; HRpeak = peak heart rate; Wmax = maximal workload; WRpeak = peak work rate; VO_2_peak = peak oxygen uptake; MHR = maximum heart rate; NR = not reported.

## Data Availability

All data analyzed in this systematic review were derived from previously published studies cited in the manuscript. The data extracted and synthesized for the review, including the study characteristics and risk-of-bias assessments, are presented in the article and Supplementary Materials. Additional extraction materials are available from the corresponding author upon reasonable request.

## Supplementary Materials

The following supporting information can be downloaded at: https://www.mdpi.com/article/10.3390/jcm15176701/s1; Table S1: Complete database-specific sensitivity-search strategies used during peer-review revision. Table S2: PRISMA 2020 Checklist.
